# MiR‐16 regulates mouse peritoneal macrophage polarization and affects T‐cell activation

**DOI:** 10.1111/jcmm.12882

**Published:** 2016-05-31

**Authors:** Xiaoqin Jia, Xiaomin Li, Yating Shen, Junjun Miao, Hao Liu, Guoli Li, Zhengbing Wang

**Affiliations:** ^1^ Department of PathologyMedical College of Yangzhou UniversityYangzhouJiangsuChina; ^2^ Department of Gastrointestinal SurgeryClinical Medical College of Yangzhou UniversityYangzhouJiangsuChina

**Keywords:** macrophage polarization, miR‐16, CD4^+^ T cells, PD‐L1

## Abstract

MiR‐16 is a tumour suppressor that is down‐regulated in certain human cancers. However, little is known on its activity in other cell types. In this study, we examined the biological significance and underlying mechanisms of miR‐16 on macrophage polarization and subsequent T‐cell activation. Mouse peritoneal macrophages were isolated and induced to undergo either M1 polarization with 100 ng/ml of interferon‐γ and 20 ng/ml of lipopolysaccharide, or M2 polarization with 20 ng/ml of interleukin (IL)‐4. The identity of polarized macrophages was determined by profiling cell‐surface markers by flow cytometry and cytokine production by ELISA. Macrophages were infected with lentivirus‐expressing miR‐16 to assess the effects of miR‐16. Effects on macrophage–T cell interactions were analysed by co‐culturing purified CD4^+^ T cells with miR‐16‐expressing peritoneal macrophages, and measuring activation marker CD69 by flow cytometry and cytokine secretion by ELISA. Bioinformatics analysis was applied to search for potential miR‐16 targets and understand its underlying mechanisms. MiR‐16‐induced M1 differentiation of mouse peritoneal macrophages from either the basal M0‐ or M2‐polarized state is indicated by the significant up‐regulation of M1 marker CD16/32, repression of M2 marker CD206 and Dectin‐1, and increased secretion of M1 cytokine IL‐12 and nitric oxide. Consistently, miR‐16‐expressing macrophages stimulate the activation of purified CD4^+^ T cells. Mechanistically, miR‐16 significantly down‐regulates the expression of PD‐L1, a critical immune suppressor that controls macrophage–T cell interaction and T‐cell activation. MiR‐16 plays an important role in shifting macrophage polarization from M2 to M1 status, and functionally activating CD4^+^ T cells. This effect is potentially mediated through the down‐regulation of immune suppressor PD‐L1.

## Introduction

Macrophages, with the capabilities of phagocytosis, antigen presentation, tissue remodelling and the secretion of a variety of molecules including growth factors, cytokines, enzymes, complement components and prostaglandins are important players in both innate and adaptive immune systems 1. Under a steady‐state or in response to inflammation, monocytes extravasate from the circulation, differentiate and mature into either dendritic cells (DCs) or macrophages 2. Mouse macrophages are characterized and distinguished from DCs by the positive expression of surface markers F4/80 and CD11b, and intracellular antigen CD68 3. When monocyte precursors exit the circulation, depending on the local microenvironment, macrophages may undergo separate differentiation pathways and generate two states of polarized activation: classically activated macrophages (M1) and alternatively activated macrophages (M2) 4, 5. These two subsets of macrophages are associated with their own *in vitro* differentiation inducers, cell‐surface markers, secretion of cytokines and other molecules, interaction with T‐cell subsets and subsequent functional consequences 6. Lipopolysaccharide (LPS) and Th1 cytokine interferon (IFN)‐γ drive macrophage polarization towards M1 phenotypes *in vitro*, which are characterized by the surface expression of CD86 and CD16/32, the secretion of pro‐inflammatory cytokines tumour necrosis factor (TNF)‐α, interleukin (IL)‐12 and IL‐23, the up‐regulation of chemokines CXCL9 and CXCL10, and the enhanced activity of inducible nitric oxide synthase (iNOS) that stimulates NO production from macrophages. Functionally, M1 macrophages are key effector cells for antigen‐specific Th1 and Th17 cellular immune responses. In contrast, M2 macrophages are differentiated in response to Th2 cytokine IL‐4, featuring the surface expression of mannose receptor (CD206), arginase 1 (Arg‐1) and Dectin‐1, the secretion of anti‐inflammatory cytokines IL‐10 and IL‐1RA, and the up‐regulation of chemokines CCL17, CCL22 and CCL24. Functionally, M2 macrophages are mainly involved in immunosuppression and tissue repair 7, 8. As a major type of infiltrating leucocytes associated with solid tumours, tumour‐associated macrophages (TAMs) play an important role in tumour immunity; featuring a IL‐10^high^ IL‐12^low^ phenotype similar to M2 macrophages and presenting potent immunosuppressive functions 5. Consistently, the predominant expression of M2 macrophages is associated with the advanced stage of tumour progression, which promotes the idea of treating cancer by the repolarization of macrophages from the immunosuppressive M2 phenotype to the pro‐inflammatory M1 phenotype 9. The polarization to M1 or M2 macrophages are highly dynamic and plastic to external signals such as the cytokine environment 10. However, intracellular mechanisms regulating macrophage polarization plasticity remains to be elucidated.

MicroRNAs (miRNAs) are small (21–25 nucleotides in length) non‐coding RNA molecules that control gene expression at post‐transcriptional levels and target more than 60% of genes in mammals 11, 12. The seed sequence of miRNAs, through base pairing with complementary sequences within the 3′‐untranslated region (3′‐UTR) of specific mRNA molecules, silences these mRNAs *via* the following mechanisms: cleavage or destabilization of target mRNA molecules (upon perfect or nearly perfect complementarity), or less efficient translation of the mRNA into proteins (for imperfect hybridization) 13, 14, 15. MiRNAs play essential roles in various physiological and pathological processes, and their biological functions and regulatory mechanisms are under intensive investigation in biomedical fields.

MiR‐16 and miR‐15a are on the same gene cluster that maps to the human chromosome 13q14 region. The down‐regulation and deletion of miR‐16 and miR‐15a has been reported in multiple cancers including chronic lymphocytic leukaemia (CLL), prostate cancer, multiple myeloma, pancreatic cancer, ovarian cancer, malignant melanoma, colorectal cancer and urinary bladder cancer 16; suggesting that the loss of these genes promote tumorigenesis. Consistently, previous studies have revealed multiple targets for miR‐16 including BCL2, CCND1 and WNT3A 17, 18, 19, 20, which are involved in tumour cell apoptosis or cell‐cycle regulation; and thus, directly regulate tumour growth. However, less is known on the action of miR‐16 in macrophage polarization, its potential targets involved in this process, or its implication in tumour development. To address these questions, we established an *in vitro* cell system, in which primary macrophages were isolated from mouse peritoneum and induced to differentiate into M1 or M2 cells in response to different cytokines. Using this model system, we were able to examine the role of miR‐16 in macrophage polarization and explore potential targets that regulate this process.

## Materials and methods

### Isolation and treatment of mouse peritoneal macrophages

All animal experiments were approved by the Institutional Animal Care and Use Committee of Yangzhou University (Yangzhou, China). Peritoneal macrophages were isolated from healthy, female C57BL/6 mice (6–8 weeks old; purchased from the College of Veterinary Medicine, Yangzhou University), as previously described 3. To characterize the purity of isolated macrophages, cells were examined after 8 hrs of isolation by flow cytometry, as detailed below.

To induce the differentiation of mouse peritoneal macrophages at 8–12 hrs after isolation, 100 ng/ml of IFN‐γ (Peprotech, Rocky Hill, NJ, USA) with 20 ng/ml of LPS (Peprotech) or 20 ng/ml of IL‐4 (Peprotech) was added to the cells and incubated at 37°C with 5% CO_2_ for 36 hrs.

### Flow cytometry

Flow cytometry analysis was performed as previously described 3 using the following antibodies: APC‐conjugated antimouse F4/80 (eBioscience, San Diego, CA, USA), APC‐conjugated antimouse CD16/32 (Biolegend, San Diego, CA, USA), APC‐conjugated antimouse CD206 (Biolegend), PE‐conjugated antimouse Dectin‐1 (Biolegend), PE‐conjugated antimouse CD4 (eBioscience) and FITC‐conjugated antimouse CD69 (eBioscience).

### ELISA

ELISA kits (Bio‐Swamp, Shanghai, China) for mouse IL‐2, IL‐4, IL‐10, IL‐12 and IFN‐γ were used to detect cytokines secreted from cells into the culture medium, according to manufacturer's instructions.

### Nitric oxide assay

Nitric oxide level in culture medium was determined using a Griess assay‐based nitric oxide detection kit (Beyotime, Jiangsu, China), according to manufacturer's instructions.

### Quantitative real‐time PCR

To determine the endogenous level of miR‐16, we performed quantitative RT‐PCR. Briefly, total RNA was extracted from cells using Trizol reagent (Invitrogen, Carlsbad, CA, USA). cDNA synthesis and miRNA quantification was achieved using the Mir‐X miRNA First‐Strand Synthesis and qRT‐PCR SYBR Kits (Takara, Mountain View, CA, USA) according to the manufacturer's instructions. The primers used were as follows: for miR‐16, forward 5′‐TAGCAGCACGTAAATATTGGCG‐3′; for U6, forward 5′‐ CTCGCTTCGGCAGCACA‐3′, and miR‐16 and U6 reverse primer was included in Mir‐X miRNA First‐Strand Synthesis Kit. All reactions were set up in triplicate, with each experiment repeated three independent times. The relative quantification in gene expression was determined using the 2^−ΔΔCt^ method 21.

### Generation and infection of lentivirus

Lentivirus‐expressing miR‐16‐1 (LV‐miR‐16) and a control lentivirus (LV‐control) containing a scrambled nucleotide sequence were generated on the enhanced green fluorescent protein (EGFP)‐expressing parental lentiviral vector GV254 by Genechem (Shanghai, China). To infect the target cells with the lentivirus, LV‐miR‐16 or LV‐control was added to the target cells with polybrene (final concentration: 5 μg/ml; Genechem). After 72 hrs, cells were imaged under a fluorescence inverted microscope (Olympus, Tokyo, Japan) for EGFP expression. Cells were used for further analysis when more than 80% of cells were GFP‐positive. For controls, cells that were not infected or those infected with LV‐control were used.

### Purification of CD4^+^ T cells from mouse spleen

To purify CD4^+^ T cells from mouse spleen, mouse spleens were dissected from healthy, female C57BL/6 mice (6–8 weeks old; purchased from the College of Veterinary Medicine, Yangzhou University) under sterile conditions. A 200‐μm cell strainer was placed in a sterile 6‐cm Petri dish and the spleens were transferred into the cell strainer with 1 ml of PBS. The spleens were mashed with a plunger from a 2‐ml syringe to release splenocytes into the Petri dish. Then, cell suspension from the Petri dish was transferred into 15‐ml conical tubes, spun down at 650 × g for 5 min. at 4°C, and the supernatant was discarded. The pellet cells were lysed in a 3‐ml RBC lysis buffer (0.15 M of NH_4_Cl, 1 mM of KHCO_3_ and 0.1 mM of ethylenediaminetetraacetic acid) at 4°C for 10 min. After washing twice with PBS, cells were incubated with PE‐conjugated antimouse CD4 at 4°C in the dark for 30 min. and sorted for CD4^+^ T cells using a flow sorter (FACS Aria; BD Biosciences, San Jose, CA, USA).

### Co‐culture of macrophages with CD4^+^ T cells

Mouse macrophages and purified CD4^+^ T cells were seeded into 6‐well plate at 2 × 10^6^ cells/well and 6 × 10^6^ cells/well, respectively. Antimouse CD3 (0.5 mg/l; eBioscience) and antimouse CD28 (0.5 mg/l; eBioscience) antibodies were also added into the co‐culture systemto stimulate the proliferation of CD4^+^T cells. After 36 hrs of co‐culture, the medium was collected, centrifuged (14,792 × g) at 4°C for 5 min. to remove cell debris and the supernatant was stored at −80°C until further use.

### Bioinformatic search for potential miR‐16 targets

Three online tools were used to identify potential miR‐16 targets: miRNA database (http://www.sanger.ac.uk/Software/Rfam/mirna/), TargetScan (http://www.targetscan.org/), and Pictar (http://pictar.mdc-berlin.de/). Targets positively predicted by all three algorithms were considered.

### Western immunoblot

Total protein was extracted using cell lysis buffer (KeyGEN, Nanjing, China) and protein concentration was measured using a BCA kit (KeyGEN), according to manufacturer's instructions. The same amount of total proteins from different samples were separated on SDS‐PAGE gels and transferred onto polyvinylidene difluoride (PVDF) membranes. Proteins were incubated with anti‐CD274/PD1L1 (Abcam, Cambridge, MA, USA) or anti‐β‐actin (internal control, KeyGEN), followed by the corresponding secondary antibodies (Beyotime). Then, the signal was developed using an ECL kit (KeyGEN) and analysed with Gel‐Pro32 software.

### Statistical analysis

Statistical analysis was performed with SPSS 17.0 software. All experiments were performed at least three independent times. Quantitative data were presented as mean ± S.D. and compared using Student's *t*‐test. A *P* value <0.05 was considered statistically significant.

## Results

### Primary peritoneal macrophages polarize to either M1 or M2 cells in response to different cytokines

**Figure 1:**
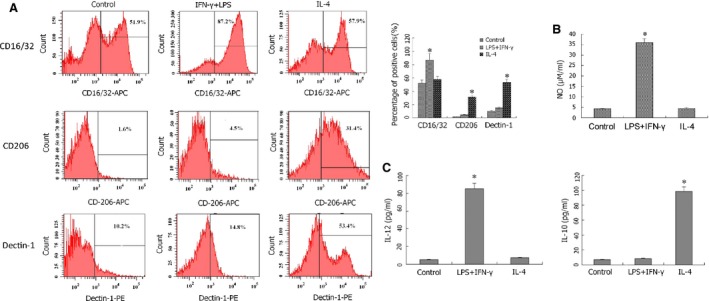
Primary peritoneal macrophages present phenotype plasticity, with the capability to be differentiated into both M1 and M2 macrophages. Primary peritoneal macrophages were isolated and treated in the absence (no treatment) or presence of IFN‐γ+LPS or IL‐4 for 36 hrs. (**A**) The expression of CD16/32, CD206 and Dectin‐1 was examined by flow cytometry and compared between the three groups. (**B**) The production of nitric oxide in medium from the three groups was measured using Griess assay. (**C**) The secretion of IL‐12 and IL‐10 in medium was determined by ELISA. *P < 0.05, compared with the other two groups.

To examine the effects of miR‐16 in macrophage polarization, an *in vitro* model system was first established; wherein, mouse primary peritoneal macrophages were isolated and induced to differentiate either into M1 cells in response to INF‐γ with LPS (INF‐γ+LPS) or M2 cells following IL‐4 treatment. Macrophages of >90% purity were obtained following a well‐established protocol, which was demonstrated by surface staining for F4/80 (Fig. S1). Without any treatment (basal state, M0), these mouse primary peritoneal macrophages contained approximately 52% CD16/32^+^ cells, <2% CD206^+^ cells and approximately 10% Dectin‐1^+^ cells (Fig. 1A). In response to INF‐γ+LPS treatment, these cells presented significant M1 features including the dramatic increase in CD16/32^+^ cells to >85%, a higher nitric oxide production, and IL‐12 secretion into the culture medium (Fig. 1B and C). In contrast, isolated peritoneal macrophages treated with IL‐4 shifted cells to prevalent M2 phenotypes with a dramatic increase in CD206^+^ and Dectin‐1^+^ cells (to >30% and >50% respectively; Fig. 1A), and a prominent IL‐10, but not NO secretion. (Fig. 1B and C).

### MiR‐16 induces M1 polarization of primary peritoneal macrophages from basal state

**Figure 2:**
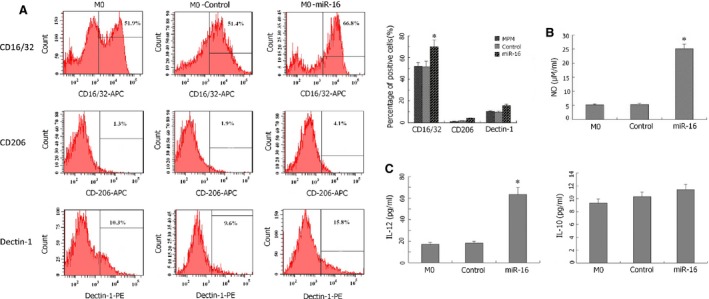
MiR‐16 shifts primary peritoneal macrophages towards M1 phenotypes. Lentivirus expression of miR‐16 was used to infect primary peritoneal macrophages (M0‐miR‐16). Non‐infected cells (M0) or control lentivirus‐infected cells (M0‐Control) were used as controls. (**A**) The expression of CD16/32, CD206 and Dectin‐1 was examined by flow cytometry and compared between the three groups. (**B**) The production of nitric oxide in medium from the three groups was measured using Griess assay. (**C**) The secretion of IL‐12 and IL‐10 in medium was determined by ELISA. *P < 0.05, compared with M0 or M0‐Control cells.

After testing the differentiation capability of isolated mouse primary peritoneal macrophages, we first examined the endogenous level of miR‐16 during the differentiation by quantitative real‐time PCR. As shown in Figure S2, the endogenous miR‐16 level was significantly lower in IL‐4‐induced M2 cells than in IFN‐γ+LPS‐induced M1 cells, suggesting that endogenous miR‐16 might be functionally important for maintaining the M1 phenotype. To assess the biological activity of miR‐16, we infected M0 cells with miR‐16‐expressing lentivirus (M0‐miR‐16); and either non‐infected parental cells (M0) or cells infected with control lentivirus (M0‐control) were used as controls. The overexpression of miR‐16 clearly shifted cells to M1 phenotypes (Fig. 2), as indicated by the increase in CD16/32^+^ cells from approximately 50% to >65% (*P* < 0.05, compared with M0 or M0‐control cells), a significantly higher secretion of nitric oxide and IL‐12 into the culture medium (*P* < 0.05, compared with M0 or M0‐control cells), and the absence of dramatic alterations on CD206^+^, Dectin‐1^+^ cells or on IL‐10 production in culture medium (*P* > 0.05, compared with M0 or M0‐control cells).

### MiR‐16 induces M1 polarization of peritoneal macrophages from M2‐polarized state

**Figure 3:**
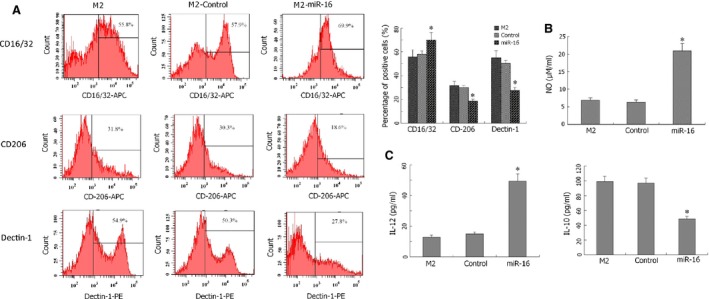
MiR‐16 skews IL‐4‐induced M2 macrophages towards M1 macrophages. Lentivirus‐expressing miR‐16 was used to infect IL‐4‐induced M2 macrophages (M2‐miR‐16). Non‐infected M2 or control lentivirus‐infected M2 cells (M2‐Control) were used as controls. (**A**) The expression of CD16/32, CD206 and Dectin‐1 was examined by flow cytometry and compared between the three groups. (**B**) The production of nitric oxide in medium from the three groups was measured using Griess assay. (**C**) The secretion of IL‐12 and IL‐10 in medium was determined by ELISA. *P < 0.05, compared with M2 or M2‐Control cells.

Next, the capacity of miR‐16 to induce M1 phenotypes on macrophages that already presented M2 features was examined. IL‐4‐treated macrophages (M2) were infected with miR‐16‐expressing lentivirus (M2‐miR‐16), and the phenotypes of these cells were compared with parental non‐infected M2 cells or M2 cells infected with control lentivirus (M2‐control). Similar to the effects on M0 peritoneal macrophages, miR‐16 induced IL‐4‐treated macrophages to shift from an M2 state to an M1 state, with a significant increase in CD16/32^+^ cells, a decrease in CD206^+^ and Dectin‐1^+^ cells, the stimulation of nitric oxide and IL‐12 production, and the repression of IL‐10 production (*P* < 0.05, compared with M2 or M2‐control cells; Fig. 3).

### MiR‐16‐expressing macrophages activate purified CD4^+^ T cells

**Figure 4:**
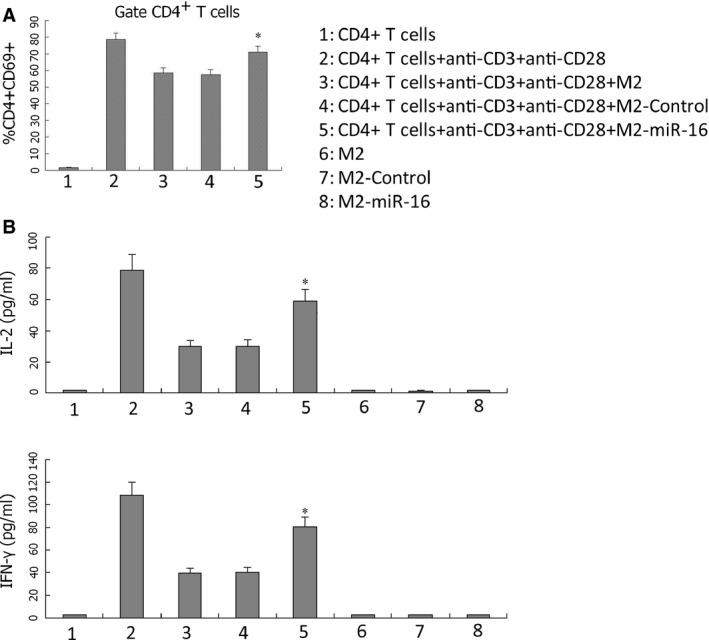
Altering miR‐16 expression in macrophages impacts CD4^+^ T‐cell activation and cytokine production. CD4^+^ T cells purified from mouse spleens were seeded onto plates in the absence or presence of anti‐CD3 and ‐CD28 antibodies, with or without M2, M2‐Control or M2‐miR‐16 cells (T:M = 3:1) for 36 hrs. (**A**) The expression of T‐cell activation marker CD69 was examined by flow cytometry, with the percentage of CD4^+^ CD69^+^ cells averaged and compared between indicated cells from at least three independent experiments. (**B**) The secretion of IFN‐γ(left) and IL‐2 (right) in culture medium was measured by ELISA. *P < 0.05, compared with the other four groups.

CD4^+^ T cells purified from mouse spleen (purity, approximately 97%; Fig. S2) were co‐cultured with M2, M2‐control or M2‐miR‐16 to analyse the functional significance of the miR‐16‐induced M1 shift. Then, anti‐CD3 and ‐CD28 antibodies were added into the co‐culture system to stimulate the activation/proliferation of CD4^+^ T cells. By quantifying cell‐surface activation marker, CD69, the addition of anti‐CD3 and ‐CD28 antibodies was found to significantly boost the activation of CD4^+^ T cells. Co‐culturing with M2 or M2‐control macrophages significantly inhibited CD4^+^ T‐cell activation, as revealed by the reduced surface expression of CD69 (*P* < 0.05, compared with CD4^+^ T+ anti‐CD3^+^ anti‐CD28 cells; Fig. 4A). In contrast, co‐culturing with M2‐miR‐16 macrophages released the suppression on CD4^+^ T‐cell activation (*P* < 0.05, compared with CD4^+^T+anti‐CD3^+^ anti‐CD28^+^ M2 or CD4^+^T+anti‐CD3^+^ anti‐CD28^+^ M2‐control); although the level of activation did not reach that achieved with anti‐CD3^+^ anti‐CD28 alone (*P* < 0.05, compared with CD4^+^T+anti‐CD3^+^ anti‐CD28 cells; Fig. 4A). Consistent with alterations in CD4^+^ T‐cell activation, the same pattern of changes was observed on the secretion of pro‐inflammatory cytokines IFN‐γ and IL‐2 in the co‐culture system (Fig. 4B). Secretions of these cytokines, which were dramatically reduced as a result of the co‐culture of CD4^+^T cells with M2 or M2‐control macrophages (*P* < 0.05, compared with CD4^+^T cells alone), were significantly released following the co‐culture of CD4^+^ T cells with M2‐miR‐16 macrophages (*P* < 0.05). In addition, when cultured with CD4^+^T, M2, M2‐control or M2‐miR‐16 alone, a minimal production of IFN‐γ or IL‐2 was detected; suggesting that cytokines detected from the co‐culture system were mostly produced by activated CD4^+^T cells.

### MiR‐16 down‐regulates PD‐L1 expression in peritoneal macrophages

**Figure 5:**
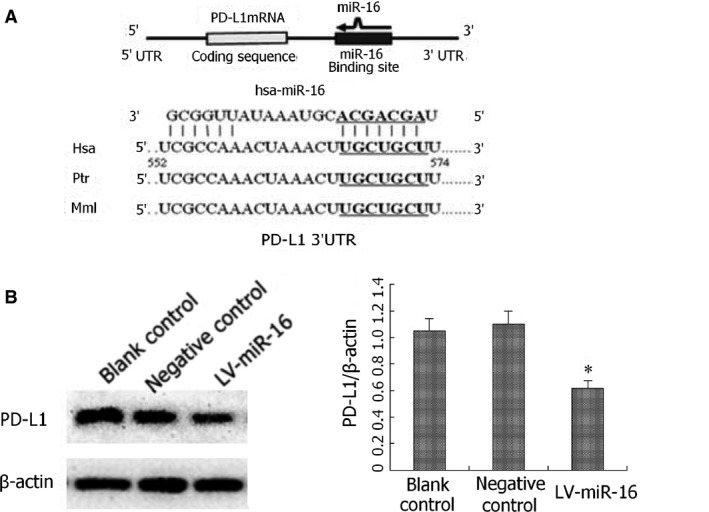
PD‐L1 is a miR‐16 target in macrophages. (**A**) Bioinformatic analysis revealed a potential miR‐16‐binding site within the 3′‐untranslated region (UTR) of PD‐L1 mRNA, which is identical for human (Has), chimpanzee (Ptr), and mouse (Mml) PD‐L1 mRNA. (**B**) The expression of PD‐L1 in M2, M2‐Control and M2‐miR‐16 cells were examined by western immunoblot, with a representative western image shown on the left and the quantification of at least three independent experiments on the right. The quantification was achieved by the signal intensity ratio of PD‐L1 to internal control (β‐actin). *P < 0.05, compared with M2 or M2‐Control cells.

Focus was given on PD‐L1, a transmembrane protein expressed on macrophages that drives the activation state of macrophages towards M2 phenotypes, to explore the potential mechanism by which miR‐16 stimulates macrophage differentiation towards M1 phenotypes 22. Through bioinformatic analysis using TargetScan, miRanda and PicTar, a potential binding site for miR‐16 within the 3′‐UTR of PD‐L1 mRNA was identified, which is identical for human, chimpanzee, and mouse PD‐L1 mRNA (Fig. 5A); suggesting that PD‐L1 could be a potential target for miR‐16. To test this possibility, the expression of PD‐L1 in M2 alone, M2‐control and M2‐miR‐16 cells were examined. Western immunoblot results revealed that PD‐L1 expression was reduced by approximately 50% in M2‐miR‐16 cells, as compared with M2 or M2‐control cells (*P* < 0.05, Fig. 5B). Consistently, we detected similar changes on the surface expression of PD‐L1 by flow cytometry (Fig. S4).

## Discussion

In this study, our seminal findings revealed that miR‐16 is sufficient to induce the differentiation of primary peritoneal macrophages or repolarize M2 macrophages towards M1 phenotypes. Molecular mechanisms underlying this miR‐16 effect involve at least the expressional regulation on PD‐L1. This study is the first to demonstrate miR‐16 action in macrophages. Furthermore, these findings would greatly impact our understanding on immune regulation, and guide the development of novel immune therapies for cancer and other immune‐related diseases.

Macrophages present great plasticity for polarization/repolarization, and several transcription factors have been suggested in regulating this process 23. However, its underlying mechanisms remain largely elusive. Darnell *et al*. found that the signal transducers and activators of transcription 1 (STAT1) homodimers induced by IFN‐γ engages the cis elements within the promoter region of target genes including iNOS and IL‐12, promotes target gene expression and drives the M1 differentiation of macrophages 24. Fujioka *et al*. revealed that although sequestered in an inactive state in quiescent monocytes, NF‐κB is induced in response to inflammatory stress; activating transcription pro‐inflammatory cytokines including TNF‐α and IL‐1β to promote M1 polarization 25. In response to IFN‐β stimulation, interferon regulatory factor 9 (IRF9) complexes with STAT2 homodimers and stimulates M1 polarization 26. Toll‐like receptor 4 signalling‐activated IRF3 enhances the production of IFN‐β, promoting the M1 phenotype 27. Interferon regulatory factor 5 is required for IL‐12 expression and contributes to M1 polarization 28. The up‐regulation of hypoxia‐inducible factor (HIF)‐1α in response to hypoxia and LPS down‐regulates Krüppel‐like factor 2, which in turn inhibits the recruitment of NF‐κB to the promoter of target genes; and thus, stimulates the M1 polarization of macrophages 29. In contrast, several other transcription factors have been shown to promote M2 polarization. HIF‐2α competes with iNOS in L‐arginine metabolism; and thus, inhibits nitric oxide production 30. Ligand‐dependent peroxisome proliferation‐activated receptor‐γ (PPAR‐γ) associates with the NCoR repressor complex to inhibit the transcriptional activity of STATs, NF‐κB and AP1; dampening M1 polarization 31. IL‐4/STAT6 signalling induces PPAR‐δ expression and promotes M2 polarization 32. Krüppel‐like factor 4 cooperates with STAT6 to mediate Arg‐1 transcription during M2 polarization 33.

In addition to transcription factors, miRNAs also modulate macrophage polarization 34, 35. Zhuang *et al*. reported aberrant miR‐223 expression in chronic inflammatory disease including rheumatoid arthritis and type 2 diabetes mellitus. MiR‐223 knockout mice, when fed with a high‐fat diet, are vulnerable to adipose inflammation and insulin resistance. In response to LPS stimulation, miR‐223 stimulates the M1 polarization of macrophages 36. Moreover, LPS up‐regulates miR‐155, which in turn inhibits transcription factor CCAAT/enhancer‐binding protein‐β (C/EBP‐β) protein expression and promotes M1 polarization 37. Consistently, C/EBP‐β is up‐regulated in TAMs, promotes M2 phenotypes in these cells and protects tumour cells form cytotoxic immunity 37. Ponomarev *et al*. revealed that brain‐specific miR‐124 is expressed in microglia, but not in monocytes or macrophages. When overexpressed in macrophages, miR‐124 inhibits M1‐macrophage polarization by inhibiting the translation of iNOS and TNF‐α, and promoting M2‐like phenotypes associated with Arg‐1 expression 38.

MiR‐16 is located at the human chromosome 13q14 region, which is within the same gene cluster as miR‐15. This gene locus is frequently deleted or mutated in multiple cancers, suggesting its tumour suppressor activity in cancer development. Accordingly, a number of miR‐16 targets have been identified to function in apoptosis 17 or cell‐cycle regulation 20, supporting the direct role of miR‐16 in tumorigenesis. In addition, miR‐16 also directly or indirectly regulates other target genes to modulate cancer behaviour and invasiveness. In leukaemia, although miR‐16 does not directly bind to the 3′‐UTR of the Wilms tumour protein 1 (WT1) mRNA, WT1 down‐regulation in response to miR‐16 significantly correlates with the development of acute myeloid leukaemia 39. In U937 lymphoma cells, miR‐16 expression is up‐regulated by LPS, which in turn negatively regulates NF‐κB signalling and stimulates IL‐8 production 40. In mammary tumour stem cells, miR‐16 negatively regulates the expression of wild‐type p53‐induced phosphatase 1 (Wip1), suppresses the self‐renewal and growth of these cells, and sensitizes breast cancer cells to chemotherapeutic agents 41. In colorectal carcinoma, miR‐15a and miR‐16‐1 directly down‐regulates the expression of AP4, a transcription factor critical for epithelial–mesenchymal transition (EMT) and cancer invasiveness/metastasis. In return, AP4 exerts a negative feedback to inhibit miR‐15a/miR‐16‐1 expression 42. To date, most studies on miR‐16 have focused on its actions in cancer cells, with minimal information available on its potential roles in other cell types within the tumour microenvironment.

In this study, we identified a novel function of miR‐16; which is its capability to promote the M1 phenotype from primary peritoneal macrophages at basal state or from IL‐4‐induced M2 macrophages. Given the importance of M2‐predominant TAM in cancer development and the loss of miR‐16 in multiple cancers, we propose that miR‐16 may also function in macrophages to complement its roles in cancer cells. To test our hypothesis, we established an *in vitro* macrophage cell system that could be induced to differentiate into either M1 or M2 phenotypes in response to distinct cytokines; and this allowed for the examination of the effects of miR‐16 in this process. Following established protocols, high‐purity macrophages (M0) were isolated from mouse peritoneum 3; and these cells were successfully polarized to either M1 or M2 phenotypes following IFN‐γ+LPS or IL‐4 stimulation 43. Isolated macrophages were infected with lentivirus to assess the significance of miR‐16 in this process, since preliminary studies that used plasmid transfection did not yield satisfactory transfection efficiency. Lentiviral infection led to the stable expression of miR‐16 in more than 80% of macrophages. In the examination of the phenotypes of these cells, surface marker expression and the production of cytokines and nitric oxide, we found that the ectopic expression of miR‐16 not only promotes the M1 polarization of primary peritoneal macrophages at basal state but also repolarizes IL‐4‐induced M2 macrophages to M1 phenotypes. This study is the first to reveal the action of miR‐16 in macrophage polarization. Interestingly, when examining the changes of endogenous miR‐16 during the differentiation, we found that it was decreased in M2 cells, as compared to M1 cells, suggesting that macrophage‐derived miR‐16 might be important for maintaining LPS+IFN‐γ‐induced M1 phenotype, but not so for the IL‐4‐induced M2 phenotype. It does not exclude the possibility that during cancer development, exogenous miR‐16 produced by other cell types such as cancer cells, may also contribute to the differentiation towards the M1 phenotype. However, the frequent deletion of the miR‐16 gene locus in many cancers would inactivate this anticancer mechanism; thus, shifting the balance to M2‐predominant phenotypes and promoting cancer development.

During the development of adaptive immunity, M1 and M2 macrophages, through antigen presentation, distinctively direct Th1 (cytotoxic) and Th2 (protective) responses respectively 44. These two responses are mainly carried out by two distinct subsets of CD4^+^ helper T cells that are divided based on the cytokines produced: Th1 cells are characterized by the secretions of IFN‐γ, IL‐1, TNF‐β and IL‐2, and participate in cellular immunity; Th2 cells mainly secrete IL‐4, IL‐6, IL‐10 and IL‐13, and regulate humoural (antibody‐mediated) immunity 45. In this study, we demonstrated that IL‐4‐differentiated M2 macrophages; and when co‐cultured with CD4^+^ T cells, inhibited the activation of the latter, which is consistent with the immunosuppressive activity of M2 macrophages. In contrast, after M2 macrophages were infected with miR‐16‐expressing lentivirus, the inhibition on CD4^+^ T‐cell activation was significantly released, which is coherent with the repolarization to the M1 phenotype in response to miR‐16 expression.

B cells are another cellular component important to T‐cell activity. It has been demonstrated that B‐cell expansion in response to antigen presentation plays a critical role in inducing T‐cell tolerance 46, 47. In addition, the study conducted by Klein *et al*. revealed that miR‐15a/16‐1 knockout is associated with B‐cell expansion and the development of CLL 48 in mice, suggesting that the up‐regulation of miR‐16 may promote B‐cell death and subsequent T‐cell activation. However, it remains unknown whether the predominant cell type in miR‐15a/16‐1 knockout mice is responsible for B‐cell expansion, or whether other genes within the locus are functionally more important than miR‐16. Future studies should characterize the cell‐specific activities of miR‐16 in various cell types, as well as abnormalities in B cells or other immune components in the context of different diseases.

MiR‐16 is highly conserved among multiple species 49. In this study, we performed a bioinformatic analysis to identify potential miR‐16 targets that carry out its actions in macrophage polarization. Through a combined search on TargetScan, miRanda and PicTar, we found a miR‐16‐binding site within the 3′‐UTR of the mouse, human and chimpanzee PD‐L1 mRNA. PD‐L1, also known as B7‐H1 and CD274, belongs to the B7 costimulatory family; and is expressed in macrophages, DCs, immune cells including activated T cells and B cells, epithelial cells and tumour cells. By interacting with its receptor programmed death‐1 (PD‐1) found on activated T cells, B cells and myeloid cells, PD‐L1 induces a co‐inhibitory signal and promote T‐cell apoptosis, anergy or functional exhaustion 50, 51. Thus, PD‐1/PD‐L1 signalling plays critical roles in regulating autoimmunity, immune responses after transplantation and cancer immunity. Consistent with their physiological functions, PD‐1 deficiency has been found to induce macrophage polarization to the M1 phenotype after spinal cord injury in mice 52. Blocking of the PD‐1/PD‐L1 pathway is currently being tested in clinic as a therapeutic approach to target cancer 53, 54. In this study, we found that the ectopic expression of miR‐16 in IL‐4‐induced M2 macrophages led to the significant reduction in PD‐L1 expression in these cells; suggesting that PD‐L1 might be a target of miR‐16 to mediate its effect on macrophage polarization. In previous studies, miRNA‐200 and miR‐513 have been reported to regulate the expression of PD‐L1 55, 56; which suggest that the expressional control of PD‐L1 might be dependent on cell types/contexts.

Although this study was carried out in isolated peritoneal macrophages, the effects observed in this study may well‐translate into *in vivo* macrophages under physiological and various pathological situations, which obviously requires further studies for its evaluation and characterization. Although miR‐16 is well‐demonstrated to be down‐regulated in multiple cancers, its expression status in TAM and potentially other stromal cells within the tumour microenvironment should be carefully examined for any potential correlation with the clinicopathological features of tumours. Mechanistically, we found a correlation between the expression of miR‐16 and PD‐L1 in macrophages; however, it remains unknown whether PD‐L1 is a direct target for miR‐16. Furthermore, it would be desirable to perform a systemic analysis on gene expression profiles in macrophages with and without miR‐16 expression, to obtain a more thorough picture of the potential gene targets of miR‐16 and functional indications of miR‐16 in addition to macrophage polarization.

In summary, we identified a novel function of miR‐16 in macrophage polarization; that is, promoting the M1 phenotype. PD‐L1 is a miR‐16 target (directly or indirectly) to mediate this process. Therefore, the significance of miR‐16 in cancer treatment might be twofold: (*i*) shifting the macrophage balance from M2‐dominant immunosuppression to M1‐mediated antitumour phenotype; (*ii*) down‐regulating PD‐L1 to block immune evasion. Given the significance of M1 and M2 macrophages in various diseases other than cancer, including autoimmunity, or resistance after transplantation, this study may provide a novel therapeutic tool for the immune regulation of various diseases.

## Conflict of interest

The authors declare no conflict of interest with this work.

## Supporting information


**Figure S1** The isolated primary peritoneal macrophages are of high purity. The purity of isolated cells from mouse peritoneum was determined by flow cytometry for F4/80+ cells (red). As a negative control, the cells were stained with isotype‐matched IgG (grey).

**Figure S2** The endogenous miR‐16 is reduced in M2 cells, when compared with M1 cells. Primary peritoneal macrophages were isolated and treated in the presence of IFN‐γ+LPS or IL‐4 for 36 hrs. The expression of miR‐16 was examined by quantitative RT‐PCR. **P < 0.01.

**Figure S3** The sorted CD4^+^ T cells from mouse spleen are of high purity. Cells isolated from mouse spleen were stained with PE‐conjugated anti‐CD4 antibody and examined by flow cytometry before (left) and after (right) sorting.

**Figure S4** PD‐L1 is down‐regulated by miR‐16 in macrophages. The surface expression of PD‐L1 in M1, M2, M2‐control and M2‐miR‐16 cells were examined by flow cytometry, with the percentage of PD‐L1^+^ cells presented and compared between different groups. *P < 0.05, compared with M2 or M2‐control cells.
