# Supplementary figures and images for: MiR‐16 regulates mouse peritoneal macrophage polarization and affects T‐cell activation
Source: J Cell Mol Med. 2016 May 31;20(10):1898–907. doi: 10.1111/jcmm.12882 (PMC5020626; doi:10.1111/jcmm.12882)

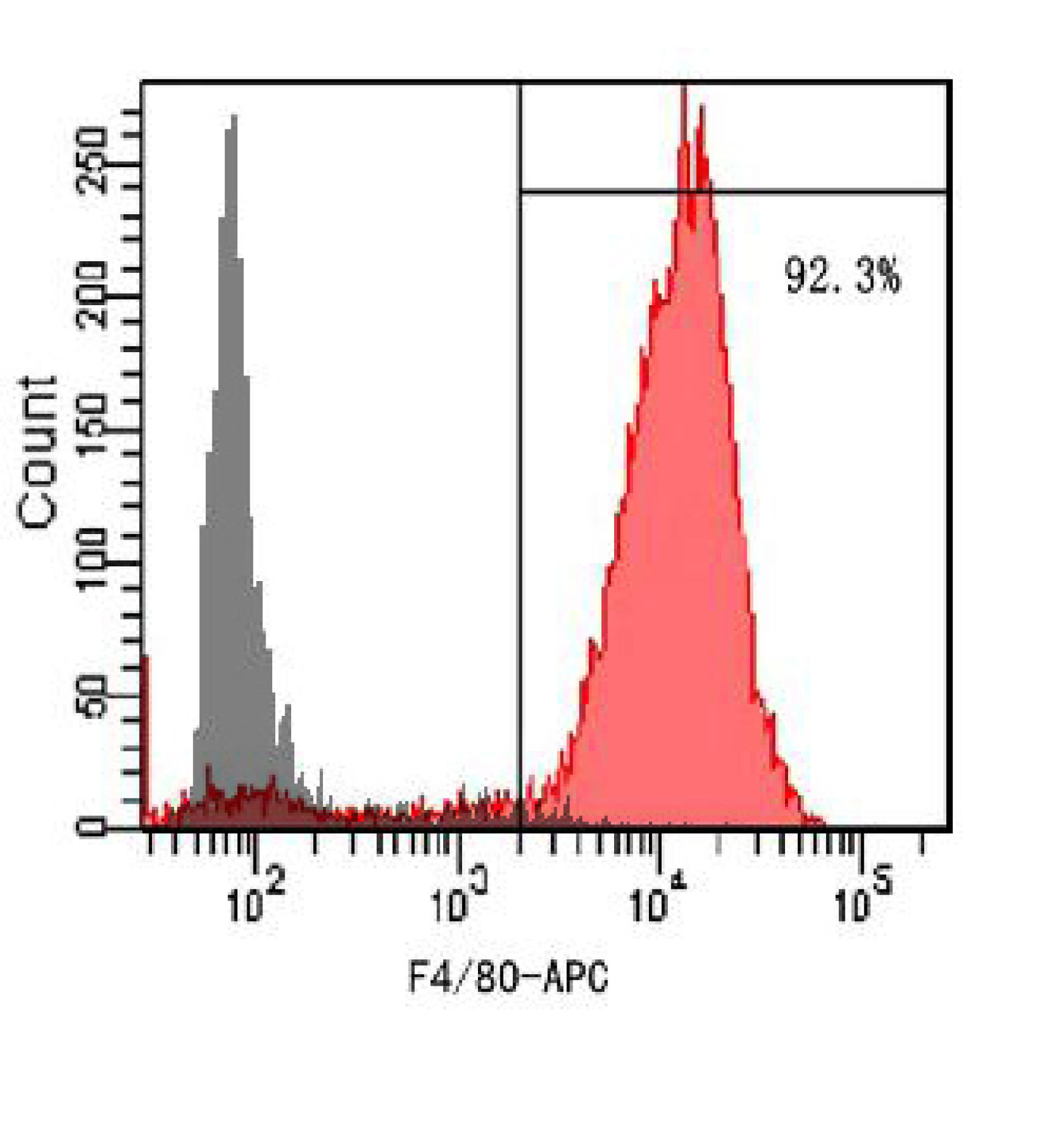

Supplement: Supplementary file 1 — Figure S1 The isolated primary peritoneal macrophages are of high purity. The purity of isolated cells from mouse peritoneum was determined by flow cytometry for F4/80+ cells (red). As a negative control, the cells were stained with isotype‐matched IgG (grey). [file JCMM-20-1898-s001.tiff]

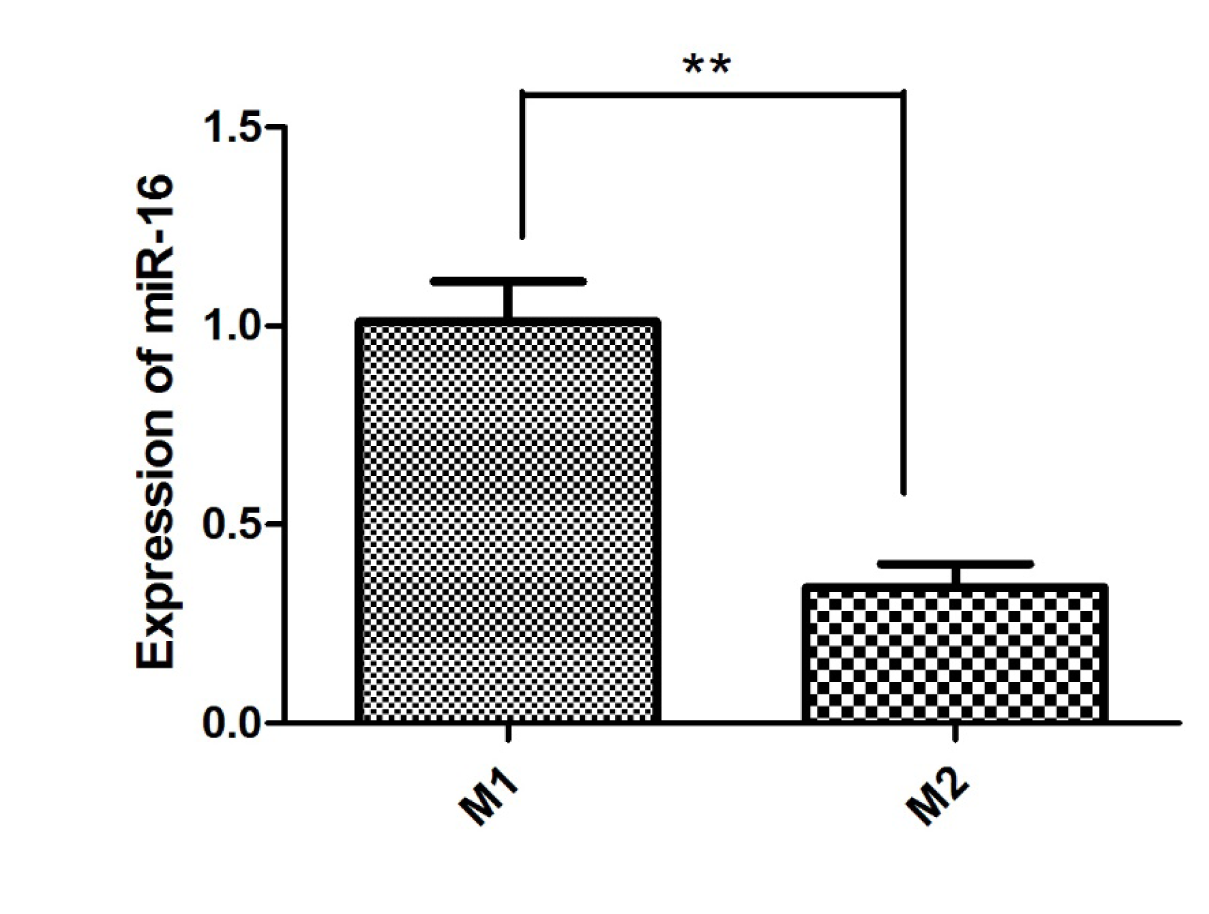

Supplement: Supplementary file 2 — Figure S2 The endogenous miR‐16 is reduced in M2 cells, when compared with M1 cells. Primary peritoneal macrophages were isolated and treated in the presence of IFN‐γ+LPS or IL‐4 for 36 hrs. The expression of miR‐16 was examined by quantitative RT‐PCR. **P < 0.01. [file JCMM-20-1898-s002.tiff]

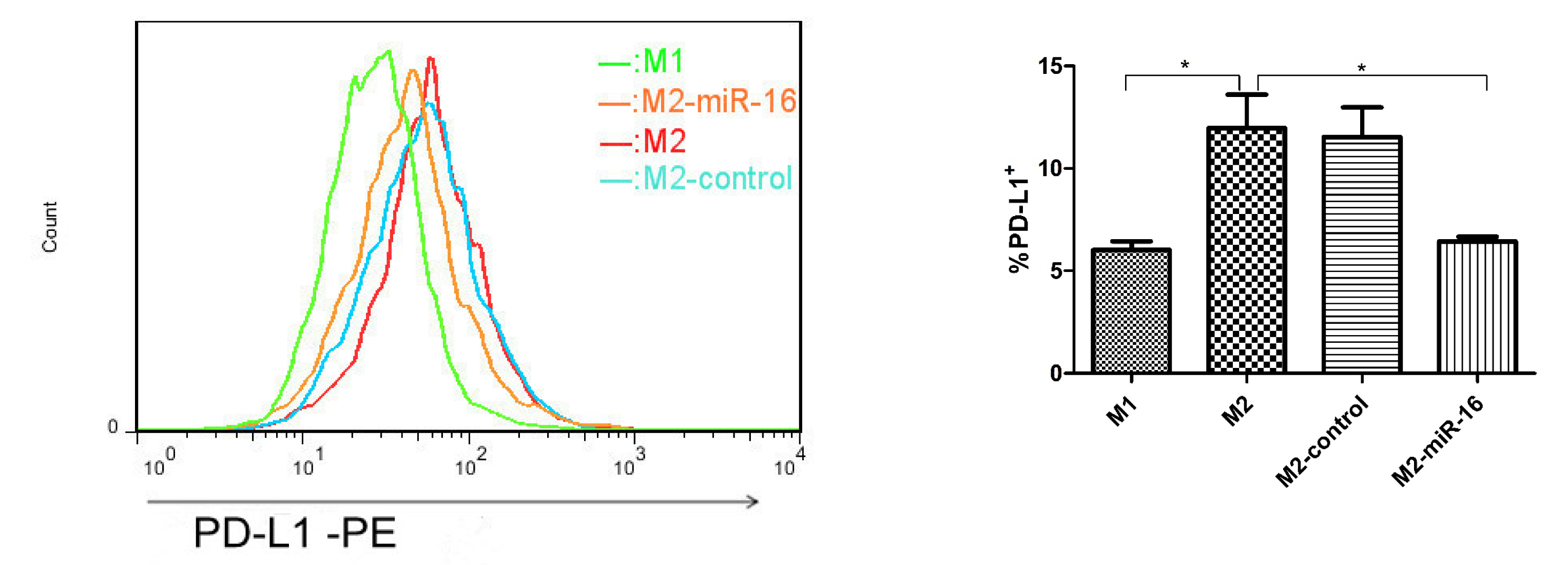

Supplement: Supplementary file 3 — Figure S3 The sorted CD4+ T cells from mouse spleen are of high purity. Cells isolated from mouse spleen were stained with PE‐conjugated anti‐CD4 antibody and examined by flow cytometry before (left) and after (right) sorting. [file JCMM-20-1898-s003.tiff]

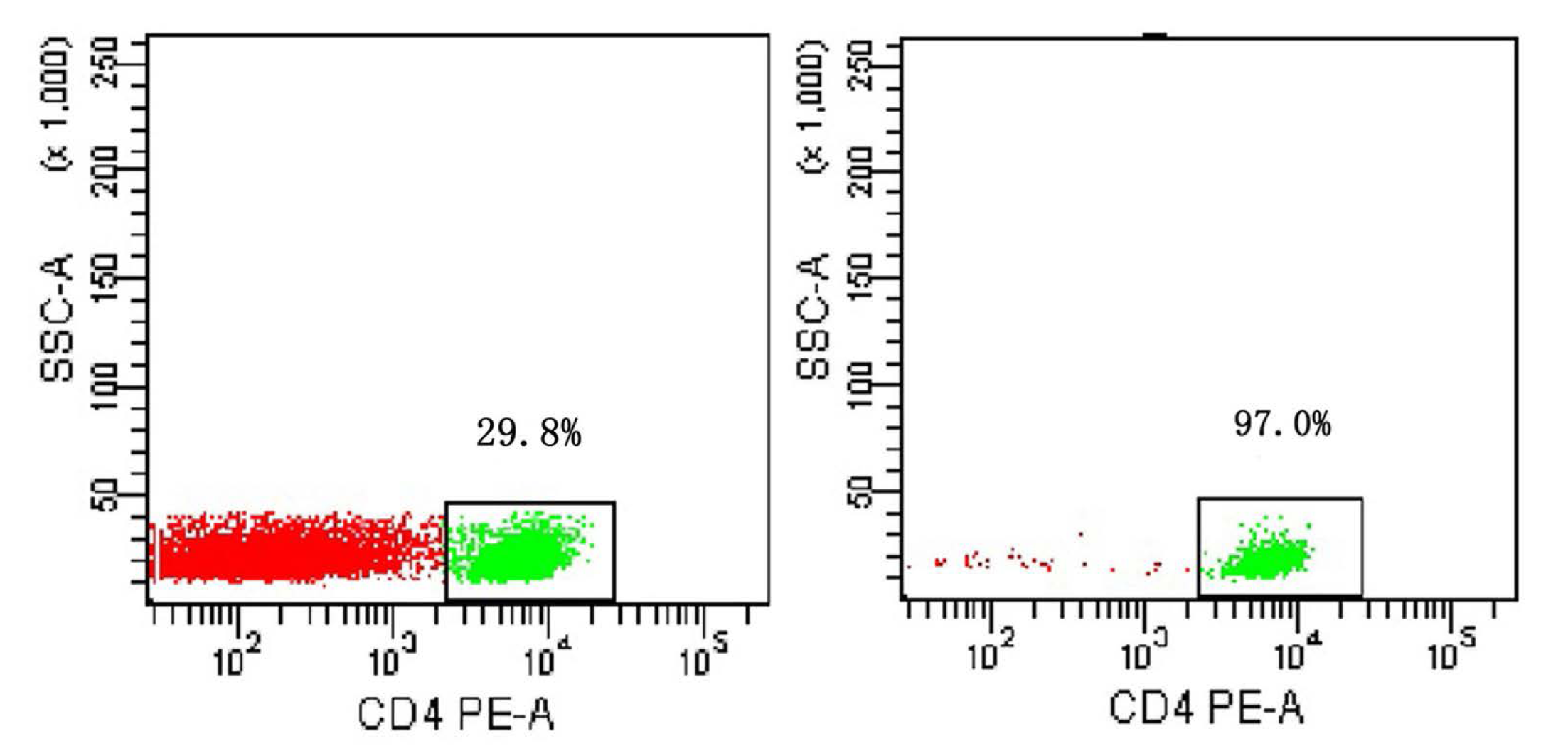

Supplement: Supplementary file 4 — Figure S4 PD‐L1 is down‐regulated by miR‐16 in macrophages. The surface expression of PD‐L1 in M1, M2, M2‐control and M2‐miR‐16 cells were examined by flow cytometry, with the percentage of PD‐L1+ cells presented and compared between different groups. *P < 0.05, compared with M2 or M2‐control cells. [file JCMM-20-1898-s004.tiff]
